# Facilitating the Cellular Accumulation of Pt-Based Chemotherapeutic Drugs

**DOI:** 10.3390/ijms19082249

**Published:** 2018-08-01

**Authors:** Ian Henry Lambert, Belinda Halling Sørensen

**Affiliations:** Department of Biology, Section of Cell Biology and Physiology, Universitetsparken 13, University of Copenhagen, 2100 Copenhagen, Denmark; belinda.sorensen@bio.ku.dk

**Keywords:** cisplatin, B12 conjugates, PEG-HAS conjugates, folate derivatives, volume-regulated anion channels, integrins

## Abstract

Cisplatin, carboplatin, and oxaliplatin are Pt-based drugs used in the chemotherapeutic eradication of cancer cells. Although most cancer patient cells initially respond well to the treatment, the clinical effectiveness declines over time as the cancer cells develop resistance to the drugs. The Pt-based drugs are accumulated via membrane-bound transporters, translocated to the nucleus, where they trigger various intracellular cell death programs through DNA interaction. Here we illustrate how resistance to Pt-based drugs, acquired through limitation in the activity/subcellular localization of canonical drug transporters, might be circumvented by the facilitated uptake of Pt-based drug complexes via nanocarriers/endocytosis or lipophilic drugs by diffusion.

## 1. Introduction

Following Rosenberg’s discovery of cisplatin back in the 1960s [1], other Pt-based drugs have been synthesized and tested for their potential anticancer activity. Today cisplatin as well as carboplatin and oxaliplatin are clinically approved for use worldwide and are the frontline choice of clinical therapy for many malignancies including lung, ovarian, testicular, and bladder cancer, melanomas, myelomas, and lymphomas. However, following an initially successful response to treatment, the efficiency of the drug is unfortunately limited due to the occurrence of severe side effects (nephrotoxicity, ototoxicity, peripheral neurotoxicity, and vomiting) together with an increased ability of the cancer cells to limit drug accumulation, repair DNA damage, and abolish intracellular cell death programs. It is emphasized that drug resistance, caused by the limitation of cellular drug accumulation via specific transporters in the plasma membrane, may also exclude alternative chemotherapeutics sharing similar uptake mechanisms. However, as illustrated in the following, camouflaging cisplatin in Vitamin B_12_ and nanocarriers or synthesizing lipophilic cisplatin analogues could be a way to facilitate cisplatin uptake and hence regain its clinical effectiveness.

## 2. Apoptotic Cell Death and Drug Resistance

Apoptosis, a cellular process during which the organism can dispose of unwanted and damaged cells, is characterized by a reduction in cell volume (AVD: apoptotic volume decrease) due to net loss of KCl and organic osmolytes, cytosolic acidification, activation of (i) tumor suppressors such as p53 that ensure the expression of p21^Waf/1Cip1^, which halts cell cycle progression, (ii) DNAases that cause DNA degradation, as well as (iii) pro-apoptotic proteins like Bax and Bid, which cause collapse of the mitochondrial transmembrane potential and release of cytochrome c. The latter recruits Apaf-1 and procaspase 9, which triggers a caspase 9/3 signaling cascade, which after the apoptotic process culminates in the formation of cellular blebs/apoptotic bodies, with the latter being phagocytosed by macrophages [2,3]. The expression of p53 is under normal conditions low due to MDM2 (murine double minute 2)-mediated ubiquitination and subsequent proteasomal p53 degradation. However, p53 phosphorylation by specific kinases, e.g., p38, following osmotic cell shrinkage, or ATM (ataxia-telangiectasia-mutated kinase)/ATR (ataxia-telangiectasia and Rad3-related) following cisplatin-induced DNA damage stabilizes p53 and hence ensures apoptosis and depresses cell proliferation. Resistance towards cisplatin can be an innate property (intrinsic resistance), associated with cell differentiation or genetic changes occurring during tumor formation or alternatively an acquired property (extrinsic resistance) that arises through proliferation of drug-resistant cells with selective advantages [4]. In general, tumorigenic cells develop resistance through (i) a reduction in cisplatin accumulation, sequestration of cisplatin in intracellular vesicles or increase in cisplatin exporting transporters, (ii) improved cisplatin detoxification or scavenging by glutathione and metalothionins, (iii) activation of DNA repair pathways that counteract the cisplatin-induced DNA inter-strand cross-links, and (iv) a shift in intracellular signaling that controls cell death programs (apoptosis, necrosis, autophagy) and proliferation. It is noted that genes encoding proapoptotic proteins, e.g., Bcl-2 and p53, can acquire mutations that lead to drug resistance through the impairment of apoptosis [5,6], whereas facilitated uptake of cytochrome c and transferrin conjugates via the transferrin receptor induces caspase 3 activation and hence apoptosis in human alveolar carcinoma cells (A549) [7].

## 3. Cellular Cisplatin Accumulation

**Figure 1:**
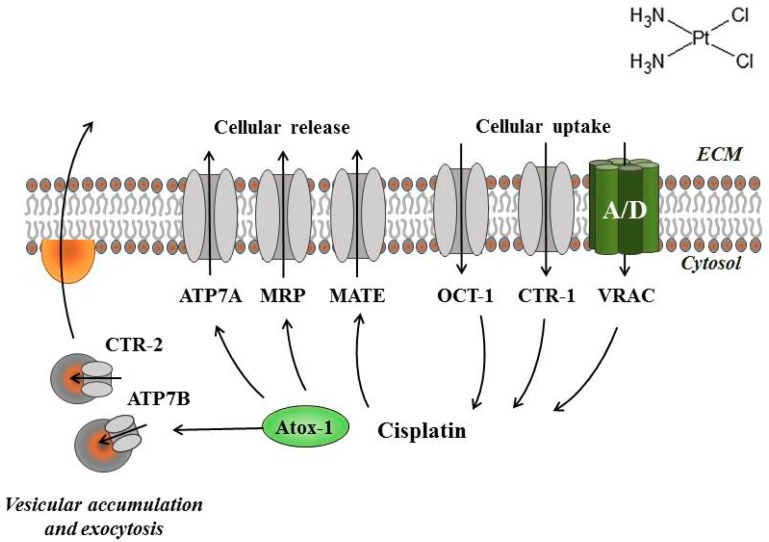
Cisplatin transporters involved in cisplatin accumulation. Cu transporters: CTR1, CTR2, ATP7A, and ATP7B. ABC transporters: MRP. Chaperone protein: Atox-1. Organic cation transporters: OCT1. Multidrug and toxin extrusion family members: MATE. Volume sensitive, LRRC8A/D-containing proteins: VRAC.

**Figure 2:**
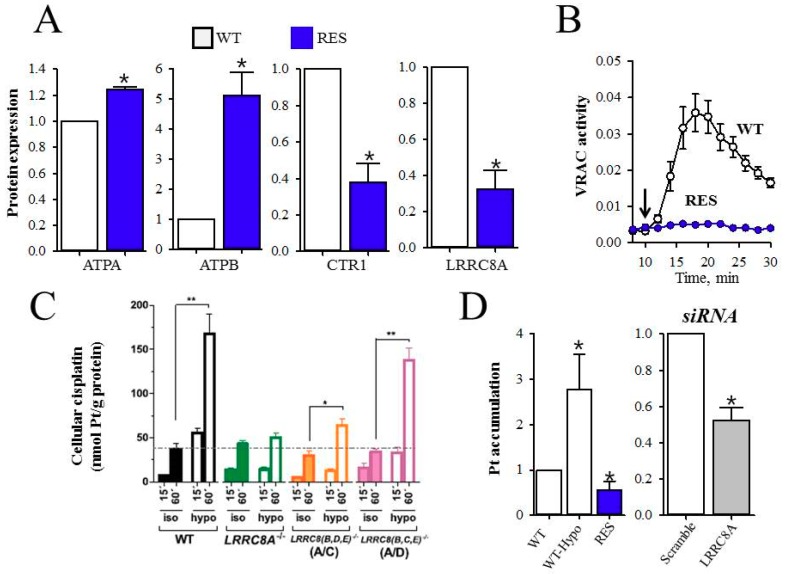
Acquired cisplatin resistance correlates with reduced VRAC activity and Pt accumulation. (**A**) Protein expression in cisplatin-sensitive (WT) and cisplatin-resistant (RES) A2780 cells was determined by SDS-PAGE/Western blot techniques. Protein expressions, initially determined relative to a house holding protein, are given relative to values from WT cells. * indicates a significant difference from WT cells. Adapted from [27]; (**B**) VRAC activity in WT and RES cells was determined by the tracer technique using [^3^H]taurine. Release of taurine is shown as the fractional rate constant (min^-1^), measured under isotonic (300 mOsm; 8–10 min) and hypotonic (200 mOsm; 10–30 min) conditions. Arrow indicates time of hypotonic challenge. Adapted from [38]; (**C**) Cellular cisplatin uptake in HEK cells following short-term hypotonic and isotonic exposure in the presence of 200 µM cisplatin. Cisplatin content was estimated as the total Pt-signal (ICP-MS) in cellular pellets. * and ** indicate significantly increased by hypotonic challenge (* *p* < 0.05; ** *p* < 0.01) Reproduced with permission from [40]; and (**D**) Pt accumulation in A2780 cells. Cellular Pt content (ng per mg protein) was determined by ICP-MS following 4 h exposure to 10 µM cisplatin in WT under isotonic and hypotonic conditions, in RES under isotonic conditions, and in WT cells treated with scramble siRNA (control for the siRNA technique) or LRRC8A siRNA. Adapted from [27]. * indicates a significant difference from WT cells and scramble siRNA.

**Figure 3:**
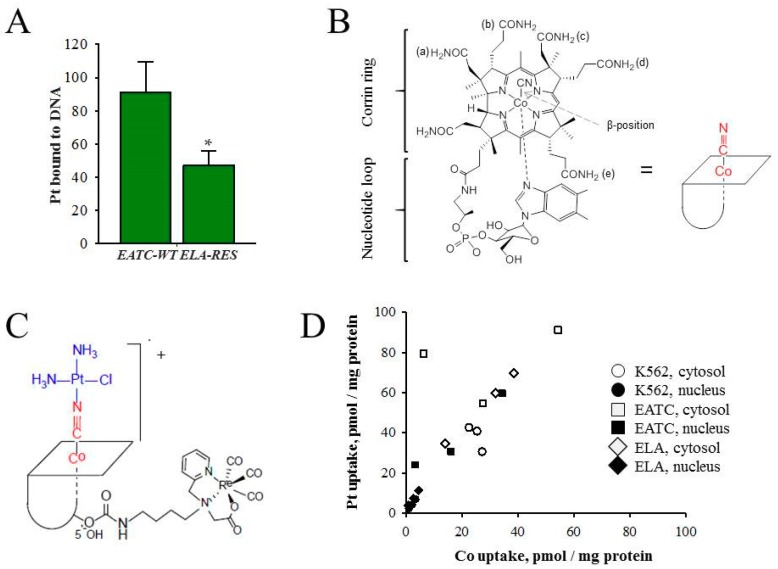
Vitamin B12 assisted cisplatin accumulation. (**A**) DNA, purified from non-adherent, cisplatin-sensitive Ehrlich cells (EATC-WT) and adherent, cisplatin-resistant Ehrlich cells (ELA-RES) following 18 h exposure to 10 µM cisplatin, was quantified and the DNA-bound cisplatin was estimated by ICP-MS. Pt content is given relative to the DNA content (pg/ng DNA). * DNA-bound cisplatin in ELA-RES significantly lower compared to EATC-WT (* *p* < 0.05). Adapted from [55]; (**B**) Vitamin B12; (**C**) [{Re}-{Co}-CN-{*cis*-PtCl(NH_3_)_2_}]^+^; and (**D**) Correlation between Pt (drug) and B12 (Co) uptake in the cytosolic and nuclear compartments in human immortalized myelogenous leukemia (K562), EATC, and ELA cells. Panels (**B**–**D**) reproduced with permission from [54].

**Figure 4:**
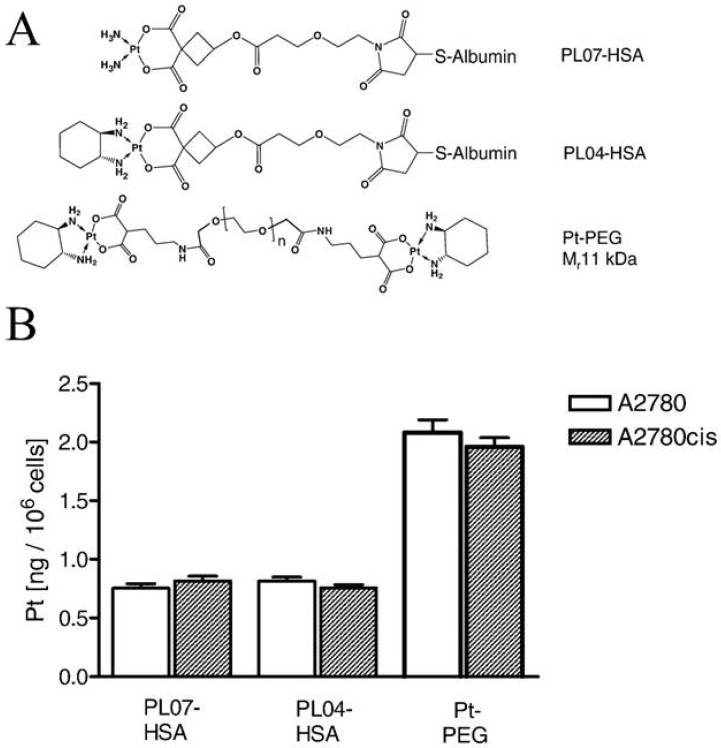
Accumulation of HAS and PEG conjugated Pt compounds in cisplatin-sensitive (A2780) and cisplatin-resistant (A2780cis) human ovarian cancer cells. (**A**) Structure of the Pt-albumin complexes PL07-HSA, PL04-HSA, and the Pt–polyethylene glycerol complex (PT-PEG); and (**B**) Platinum accumulation after 120 min exposure to PL07-HSA, PL04-HSA and PT-PEG. Reproduced with permission from [63].

**Figure 5:**
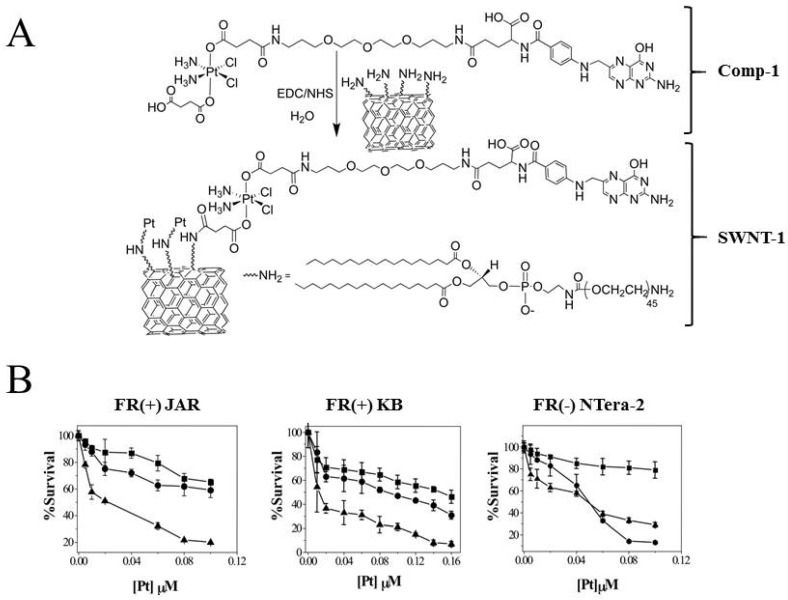
Cytotoxicity of Pt- compounds attached to nanotubes. (**A**) *c*,*c*,*t*-[Pt(NH_3_)_2_Cl_2_(O_2_CCH_2_CH_2_CO_2_H) (O_2_CCH_2_CH_2_CONH-PEG-FA)] (Comp-1) is attached to the surface of the carbon nanotube (SWNT-PL-PEG-NH_2_) forming SWNT-1; and (**B**) Survival of human nasopharyngeal epidermoid carcinoma (KB), choriocarcinoma (JAR), and human testicular cancer (NTera-2) cells treated with Comp-1 (square), SWNT-1 (triangle), or cisplatin (circle) at various concentrations for 72 tested by a MTT assay. Reproduced with permission from [71].

**Figure 6:**
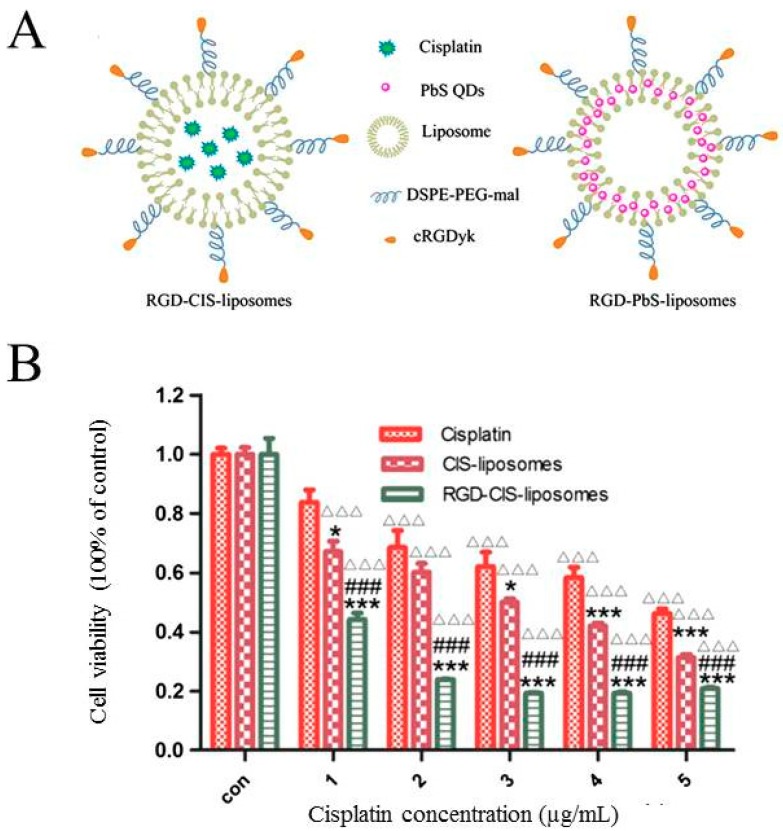
Multifunctional liposomes. (**A**) Schematic diagram of cisplatin (RGD-CIS) and Pbs (RGD-PbS) liposomes. cRGDyk is the integrin ligand. DSPE-PER-mal is 1,2-distearoyl-sn-glycerol-3-phosphotehanolamine-*N*-[maleimide(polyethylene glycol)-2000]; and (**B**) In vitro cytotoxicity (MTT-assay, RM-1 cells) after 48 h exposure to cisplatin, CIS-liposomes, and RGD-CIS-liposomes. * *p* < 0.05, *** *p* < 0.001 versus cisplatin; ### *p* < 0.001 versus CIS-liposomes; △△△ *p* < 0.001 versus control. Reproduced with permission from [77].

Limitations in intracellular drug accumulation will inevitably cause cisplatin resistance. Figure 1 and Figure 2 reveal transporters facilitating cisplatin uptake/release across the plasma membrane in cisplatin-sensitive cancer cells, whereas Figure 3, Figure 4, Figure 5 and Figure 6 illustrate how we, through incorporation of cisplatin in larger complexes, can benefit from the activity of alternative transporters/receptors and hence ensure the uptake of cisplatin in cancer cells, where resistance is caused by an otherwise impaired cisplatin accumulation.

### 3.1. Copper Transporters and ATPases

The copper transporters CTR1 and CTR2, which we normally associate with the cellular accumulation of Cu ions, have for a long time been considered important facilitators of cellular cisplatin accumulation. The functional CTR1 transporter is a homo-trimer, where each monomer has three trans-membrane domains with C-terminals exposed to the cytosol [8]. It appears that loss of the labile chloride ligands allows cisplatin to interact with methionine residues, which normally guide Cu ions through the CTR1 pore through trans-chelation [9]. Furthermore, cisplatin, once on the intracellular site of the membrane, is reported to bind to a potential phosphorylation site (Tyr^103^) involved in CTR1 endocytosis and Cys^189^ close to the C-terminal, which is coupled to correct assembly of the CTR1 trimer in the plasma membrane [10]. Cisplatin accumulation is reduced following downregulation of CTR1 [11] and in humans it has been shown that cisplatin causes a rapid degradation of CTR1, diminishing cisplatin uptake and prompting cisplatin resistance [12]. Genetic CTR1 knockout induces cellular cisplatin resistance in vivo, whereas overexpression of CTR1 has been shown to correlate with increased cisplatin accumulation and sensitivity [12]. In a preclinical study, it has been shown that inhibition of proteasomal degradation using bortezomib prevented cisplatin-induced downregulation of CTR1 in ovarian cancer cells, thereby causing an increased cisplatin accumulation and cytotoxicity [13]. CTR2 belongs to the same family as CTR1 and facilitates cisplatin uptake in endosomes and macro-pinocytosis through the activation of, e.g., small GTPase (Rac1) and the cell division control protein 42 homolog (cdc42) [14]. It has been suggested that knockdown of CTR2, i.e., limitations in cellular cisplatin export, could be a strategy to overcome cisplatin resistance [14]. However, it has to be noted that the role of CTR1/CTR2 in facilitated cisplatin uptake has been questioned as genomic knockout (Crisp-Cas9) does not affect cisplatin sensitivity in human HEK-2931 and ovarian carcinoma cells [15].

ATP7A and ATP7B are ATPases that together with the Cu chaperone antioxidant 1 (Atox1) facilitate Cu export, and it has been demonstrated that the ATP-driven movement of Cu- or Pt-related charge through ATP7A/B involves binding to CXXC motifs located at the cytosolic, N-terminal metal binding domains of the transporters [16]. Using cisplatin-sensitive and cisplatin-resistant human ovarian cancer cells (A2780), Kalayda and co-workers have shown that ATP7A/ATP7B mainly localize to the trans-Golgi network in drug-sensitive cells, whereas they seem to become more sequestrated to peripheral vesicular structures in resistant cells [17]. It has, however, turned out that ATP7A and ATP7B also play a role in sensitivity to platinum drugs as they mediate the efflux and/or sequestration of drugs in sub-cellular compartments [17,18,19,20,21] and ATP7A/ATP7B trafficking to the plasma membrane increases following an increase in Cu or cisplatin [17,22]. Furthermore, ATP7A/ATP7B expression is upregulated in cisplatin-resistant cancer cell lines and overexpression correlates with the cisplatin-resistant phenotype [12]. In congruence, Wang and co-workers indicated that cisplatin resistance in vincristine-resistant Hep-2v cells correlated with high levels of ATP7B [23]. Furthermore, they demonstrated that exogenous miR-133a, which through induction of apoptosis and inhibition of tumor cell metastasis functions as a tumor inhibitor [24], reduced ATP7B expression significantly in HEP-2v cells and concomitantly lowered cell viability after cisplatin treatment [23]. Recently, Zhu and co-workers demonstrated that ATP7A deletion in H-RAS transformed tumorigenic mouse fibroblasts not only increased cellular Cu accumulation and sensitivity to stress (hypoxia, reactive oxygen species) but also cisplatin sensitivity in vitro and in vivo [25]. In another approach, it was demonstrated that the overexpression of annexin A4, which promotes cisplatin resistance through regulation of ATP7A, reduced cellular cisplatin in non-small cell lung cancer cells (A549), whereas downregulation of annexin A4 expression, using the inhibitor toosendanin, increased the cellular concentration [26]. Hence, downregulation of ATPB7/ATP7B expression/activity in combination with cisplatin could be a means to enhance cisplatin sensitivity.

The Organic Cation Transporter 1 and 2 (OCT1/2), multidrug and toxin extrusion protein 1-3 (MATE1-3), and multidrug resistance-associated protein 1/2 (MRP1/2) have also been assigned a role in the accumulation and release of cisplatin, but their influence on resistance is not fully understood [27]. However, with respect to MRP1 and MRP4 it has been demonstrated that increased expression and altered N-linked glycosylation in the oxaliplatin-resistant human ovarian carcinoma IGROV-1 cell line are associated with reduced accumulation of the platinum drugs and resistance to oxaliplatin and cisplatin [28].

### 3.2. The Volume-Regulated Anion Channel (VRAC) Family

Volume-regulated anion transporters for anions and organic osmolytes, now in general referred to as VRAC, are not only crucial for the restoration of the cell volume and cellular physiological conditions following osmotic cell swelling, but also essential for cell cycle progression, proliferation, cell migration, and instigation of apoptosis [29,30]. The intracellular signaling, prompted by osmotic cell swelling and leading to VRAC activation, as well as the kinetics for VRAC activation/inactivation have been described over the decades using the patch clamp technique (anion current), colometric titration (net transport of anions), the HPLC technique (net transport of organic osmolytes), and the radiotracer technique (unidirectional transport of ions and organic osmolytes), combined with a broad specter of pharmacological inhibitors [31,32]. However, it was only in 2014 that members of the leucine-rich repeat-containing 8 (LRRC8) protein family were first identified as being responsible for VRAC activity, i.e., for the volume-sensitive chloride current (I_Cl,swell_) and swelling-induced loss of neurotransmitters and organic osmolytes [33,34,35,36,37,38,39]. Interestingly, it has now been demonstrated that VRACs also facilitate the accumulation of cisplatin and carboplatin, but not oxaliplatin [27,40].

The LRRC8 protein family embraces the five members LRRC8A/B/C/D/E, which are all membrane-spanning proteins composed of four trans-membrane domains and a long C-terminal leucine-rich repeat domain (LRRD) [40,41]. As sequence analyses indicated that the LRRC8 family are related to pannexins’ channel complexes, which release larger signaling molecules as ATP from the cytoplasm to the extracellular environment [42,43], it has been suggested that LRRC8 proteins may form multimeric channel complexes with varying LRRC8 protein stoichiometry [41,42]. Protein sequence analysis has revealed that LRRC8A contains putative cisplatin-interacting methionines in the cellular and transmembrane segments as well as CxxC/YxxY motifs that can be oxidized/phosphorylated, but their significance for the facilitation of cisplatin uptake has yet to be revealed. Using small-interfering RNA (siRNA) (knockdown; KD) or genomic disruption (knockout; KO) of LRRC8A, Qiu et al. [35] and Voss et al. [37] demonstrated that the LRRC8A protein is essential for activation of anions, the I_Cl,swell_, and the release of organic osmolytes. Moreover, swelling- and ATP-induced release of d-aspartate (used as a non-metabolizable glutamate analogue) and taurine (used as an inert representative of organic osmolytes) in primary rat astrocytes release is abrogated when LRRC8A was knocked down [44]. It has turned out that the substrate specificity of VRAC is dictated by the combination/stoichiometry of LRRC8A with other members of the LRRC8 family, i.e., transport of the uncharged myo-inositol or the zwitterionic taurine, lysine, and GABA, depends on the presence of LRRC8A/D, whereas the transport of halides (Cl^−^, I) depends on LRRC8A/E, LRRC8A/C, and to some degree LRRC8A/D [33,40,45,46]. It is emphasized that a shift in substrate charge, i.e., shifting pH from a physiological value (pH 7.4), where most of the zwitterion taurine molecules are electroneutral, to an alkaline value (pH 9.8), where about 91% of taurine molecules are negatively charged, not only increased the VRAC transport capacity, but also eliminated the sensitivity of swelling-induced taurine release to LRRC8D knockdown [46]. This indicates that a switch in substrate charge diverts substrate movement from a LRRC8A/D-dependent VRAC to another LRRC8A-dependent, but LRRC8D-independent VRAC complex.

The data in Figure 2, obtained with human ovarian cancer (A2780) cells, indicate that cisplatin resistance correlates not only with a reduced expression of CTR1 and increased expression of ATPA and ATPB, which in itself will reduce Pt accumulation, but also a significant reduction in LRRC8A protein expression (Figure 2A). The latter data correlate with reduced VRAC activity, which is seen as an impaired, swelling-induced release of taurine (Figure 2B). Planells-Cases and co-workers demonstrated that the accumulation of Pt in human embryonic kidney (HEK) cells is dramatically increased under hypotonic conditions, but reduced by siRNA-mediated knockdown of LRRC8A and LRRC8D (Figure 2C). In A2780 cells, osmotic cell swelling likewise increases Pt accumulation, which is, however, abrogated in cisplatin-resistant cells and following LRRC8A KD (Figure 2D). Hence, a LRRC8A/D-containing VRAC complex facilitates cisplatin accumulation. Downregulation of VRAC activity as a phenotypic characteristic has been reported from several cisplatin-resistant cell lines including mouse Ehrlich ascites tumor cells (EATC) [47], human epidermoid KCP-4 cancer cells [48], human lung epithelial cancer (A549) cells [39,49], and the human promyelogenous leukemia cell line (HL60) [50]. It is noticed that cisplatin-resistant A549 cells, similar to resistant A2780 cells, have reduced swelling-induced VRAC activity, which, however, does not reflect a reduction in total LRRC8A expression, but more likely a concomitant reduction in LRRC8A expressed in the plasma membrane [39]. It is emphasized that pharmacological inhibition of the LRRC8A/D-dependent VRAC and siRNA-mediated knockdown of LRRC8A not only markedly reduce cisplatin accumulation, but also prevent intracellular cisplatin-induced apoptotic signaling, i.e., phosphorylation/activation of pro-apoptotic transcription factor p53, murine double minute-2 (MDM2) and the cell cycle restrictor p21^Waf1/Cip1^, as well as caspase-9/-3 cleavage in human ovarian A2780 and human alveolar A549 cancer cells [39]. Cells in vitro are normally exposed weekly to cisplatin to maintain the acquired phenotype; however, abrogation of cisplatin treatment not only restores VRAC activity and LRRC8A protein expression within three to six weeks in A2780 cells, but also cisplatin toxicity, i.e., the ability of cisplatin to activate the pro-apoptotic transcription factor p53, the cell cycle inhibitor p21^Waf1/Cip1^, and apoptosis (Noxa and caspase-9) [39]. Hence, characterization of the time-frame for reactivation of VRAC activity and cisplatin-induced cell death signaling could be taken into consideration to circumvent the acquisition of cisplatin resistance caused by VRAC inactivation.

A strategy to improve Pt uptake in cells could be the overexpression of LRRC8A. However, in HeLa cells LRRC8A overexpression actually decreases VRAC activity [37]. Furthermore, various forms of stress including adaptation to hypotonic conditions, exposure to ROS, or cisplatin hampering VRAC activity—not through a reduction of the LRRC8A protein expression, which is actually increased, but more likely through the modulation of the activity of kinases/phosphatases controlling VRAC activity. We speculate about whether the reduced VRAC activity, following stress-induced overexpression of LRRC8A, is a pro-survival mechanism that protects cells from excess drug uptake, AVD, and hence instigation of apoptosis. Planells-Cases and co-workers, using genome-wide screening, identified LRRC8A and LRRC8D as mediators of carboplatin and cisplatin uptake and resistance in haploid KBM7 cells [40]. Furthermore, they found that a low expression of LRRC8D, not LRRC8A, was correlated with shorter survival of ovarian cancer patients treated with platinum-based drugs [40]. In summary, decreased cellular cisplatin accumulation in resistant cells can reflect reduced uptake through CTR1 and LRRC8A/8D-dependent VRACs, as well as increased cisplatin release through MRP, and the ATPases ATP7A and ATP7B. As reversible downregulation of VRAC activity may in some cell lines involve alteration of the membrane and/or total expression of LRRC8A/LRRC8D, our current knowledge to the cellular signaling involved in the regulation of VRAC activity [31,32,38,39] and the recent revelation of the LRRC8A structure using high-resolution cryo-electron microscopy [41] raises the possibility of controlling cisplatin uptake through the manipulation of the selectivity/open-probability of the VRAC complex.

## 4. Alternative Pathways for Accumulation of Cisplatin

Simultaneous administration of two or more chemotherapeutic drugs with different mechanisms of action has been applied as the primary choice of chemotherapeutic treatment regimen, as combination therapy minimizes the development of drug resistance by targeting different signal-transduction pathways [51]. To improve the efficiency of chemotherapy, cisplatin, oxaliplatin, and carboplatin are often used in combination with either paclitaxel, docetaxel, doxorubicin (DOX), fluorouracil (5-FU), or gemcitabine [51]. However, one of the main challenges of combination therapy is that the drug ratio required for proper synergic effect of the drugs in vitro may not necessarily correlate with the same synergic effect in vivo, simply because the administrated drugs may have different metabolic profiles. Using nanocarriers for the delivery of two or more chemotherapeutic drugs gives the possibility of conjugating/encapsulating the drugs at a desired drug ratio, protecting drugs against premature degradation, reducing systemic toxicity, increasing blood retention time, and targeting cancer cells via their cellular characteristics (receptors) [51]. Moreover, nanocarriers might solve problems of poor drug solubility. Application of nanocarriers for Pt-based combination therapy has recently been reviewed in [51,52,53].

### 4.1. B12 Conjugates

As cisplatin resistance reflects a reduced accumulation of cisplatin via “conventional transporters”, alternative uptake mechanisms have been considered to regain cisplatin sensitivity in resistant cells. Various platinum-(II) and platinum-(IV) agents have been loaded/conjugated into a large variety of lipid, polymeric, inorganic nanocarriers, which include liposomes, nanoparticles, and nanotubes [51,52]. Using cisplatin-sensitive Ehrlich ascites tumor cells (EATC) and their cisplatin-resistant counterpart, Ehrlich Lettré ascites cells (ELA), Tran and co-workers tested vitamin B12 (cyano-cob(III)alamin) as a drug carrier for cisplatin [54]. Cisplatin-induced activation of apoptosis, verified by the caspase 3 activity, is almost impaired in ELA cells compared to EATC, partly due to reduced cytosolic/nuclear Pt accumulation [55] and a concomitant reduction in Pt binding to DNA (Figure 3A). B12 is an essential, water-soluble and non-toxic vitamin that plays a role in the methionine synthesis and the tricarboxylic acid cycle. B12 is an octahedral Co^III^ complex with a tetradentate corrin ring and a CN^−^ ligand in the β-axial position (Figure 3B). An advantage of B12 is that it is protected from degradation through its binding to various proteins inside living systems, e.g., haptocorrin (HC) in saliva/gastric fluids, intrinsic factor (IF), secreted by parietal cells, and transcobalamin (TC), which is present in the plasma [56,57].

Furthermore, B12 not only protects protein-peptide drugs from hydrolysis in the acidic condition of the gastrointestinal tract [58,59,60,61], but binding of B12 to TC also facilitates B12–TC complex uptake via the TC receptor or CD320 [57]. As tissue B12 accumulation increased 350% upon overdosing of the vitamin [62], the use of B12 as a drug carrier could ensure a certain transport capacity. Constructing a positively charged [{Re}-{Co}-CN-{*cis*-PtCl(NH_3_)_2_}]^+^ complex, consisting of a {fac-Re(CO)_3_}^+^ and a [*cis*-PtCl(OH_2_)(NH_3_)_2_]^+^ moiety conjugated to the B12 structure (Figure 3C), Tran and co-workers showed that, despite the chemical modification, the compound retained good affinity to all B12 transport proteins [54]. Furthermore, as the metals (Co, Re, Pt) in the B12-drug conjugate could be measured by ICP-MS (Inductively Coupled Plasma Mass Spectrometry) (^59^Co, ^195^Pt, and ^187^Re) it was shown that [{Re}-{Co}-CN-{*cis*-PtCl(NH_3_)_2_}]^+^ not only accumulated in the cytosol/nucleus of EATC, ELA, and human immortalized myelogenous leukemia (K562) cells (Figure 3D), but Pt was also bound to DNA and no substantial Pt was released from the B12 structure to the extracellular compartment [54]. It should be noticed that the positively charged [{Re}-{Co}-CN-{*cis*-PtCl(NH_3_)_2_}]^+^ was preferentially taken up as compared to native B12 [54] and that the cytosolic and nuclear Pt accumulation was always 1.6-fold higher than Co (Figure 3D). The interpretation of the data was that an additional uptake pathway for charged B12 derivatives operates in parallel to the TC-mediated B12 receptor and that a more efficient efflux of Co occurs compared to platinum [54].

### 4.2. Polyethylene (PEG) and Human Albumin (HSA) Conjugates

Garmann and co-workers investigated the cellular accumulation and cytotoxicity of two Pt-albumin complexes (PLO4-HSA, PLO7-HSA) and one Pt-polyethylene glycol complex (PEG_10k_-(Mal-Pt-DACH)_2_) (Figure 4A) in cisplatin-sensitive and -resistant A2780 ovarian cancer cells [63]. It was demonstrated that the cellular accumulation of the complexes was unaffected by the cisplatin-resistant phenotype (Figure 4B) but decreased by the presence of bafilomycin A1 [63], indicating that the complexes mainly enter the cells via endocytosis. It is noted that the PEG_10k_-(Mal-Pt-DACH)_2_ complex exhibited a significantly higher Pt-DNA adduct formation and cytotoxic activity compared to the two albumin conjugates, which could reflect that two Pt moieties are incorporated in the PEG complex [63]. Unfortunately, PEG_10k_-(Mal-Pt-DACH)_2_ also showed the highest cross-resistance to cisplatin [63]. In this context it is noted that Zhang and co-workers demonstrated that the administration of nanoparticles by FDA-approved poly (lactic-co-glycolic acid)-poly (-ethylene glycol (PLGA-PEG) that contained cisplatin, wortmannin (inhibitor of growth factor-induced proliferation), and a DNA-repair inhibitor reversed platinum resistance in A2780 cells [64].

### 4.3. Liposome Conjugates

Preclinical studies of a liposomal formulation of cisplatin have shown promising effects against ovarian, pancreatic, head and neck cancer, non-small cell lung cancer (NSCLC), and HER-2/neu–negative metastatic breast cancer [53,65,66,67,68]. In ovarian cancer, lipoplatin (liposomal cisplatin) was found to exhibit anti-tumor activity by causing caspase activation and hence apoptosis [65]. Moreover, lipoplatin was found to hinder the enzymatic activity of thioredoxin reductase (oxidative stress), reduce epidermal growth factor receptor (EGFR) expression, and inhibit cell invasion [65]. In ovarian cell lines, lipoplatin provided a synergistic effect when used in combination with doxorubicin and the albumin-bound paclitaxel [65]. It is noted that exposure of ovarian cancer cells to lipoplatin decreased aldehyde dehydrogenase and prominin-1 (also known as CD133) expression, which are markers of ovarian cancer stem cells and decreased spheroid growth and cell migration [65]. Most importantly, treatment with lipoplatin was demonstrated to inhibit tumor xenograft growth with negligible systemic toxicity, and after the treatment no tumor progression was observed in the xenograft model [65].

Besides the above examples, the nanocarrier delivery system also selectively delivers Pt-based drugs to tumor cells either by taking advantage of an enhanced permeability and retention effect (termed the EPR effect) or specific recognition between the nanocarrier and the tumor cell [51]. Selective recognition and targeting to the tumor tissue requires identification of distinct biomarkers [52,69]. Tumor biomarkers include proteins or receptors that are abnormally expressed in the plasma membrane of the tumor cell, e.g., the vascular endothelial growth factor receptor (VEGFR), the epidermal growth factor receptors (EGFR and HER2), interleukin-4 (IL-4), transferrin, and the folate receptor (FOLR) [52]. Covalent attachment of monoclonal antibodies, raised against these biomarkers, to the nanocarrier may assist with specific tumor recognition and targeting [52]. It is assumed that Pt-loaded nanocarriers are recognized and attached to receptors/biomarkers present on the cell surface of the cancer cell and taken up to a higher extent compared to the normal tissue by cancer cells through receptor-mediated endocytosis. Inside the cancer cell the Pt-drug is released into the cytoplasm via a mechanism that often involves low-pH-triggered nanocarrier disassembly and endosomal escape. Once released, the Pt-drug accumulates within the nucleus, where it binds to the DNA, causing DNA damage and instigation of the intrinsic apoptotic pathway.

### 4.4. Folate Derivatives

The folate receptor (FR) is overexpressed in the plasma membrane of a variety of human tumor cells, and FOLR-1 has in several studies been targeted by the use of platinum-folate complexes [69]. In early studies carboplatin was modified with a folate-targeted PEG (FT-PEG) construct such that it efficiently entered a murine lung carcinoma cell line (M109) through folate receptor-mediated endocytosis (FRME) [70]. Even though the FT-PEG conjugates were efficiently taken up by the M109 cells, the conjugates seemed to form fewer DNA adducts and had significantly higher IC_50_ values compared to the carboplatin control. This reduced efficiency indicates that FT-PEG conjugates may not be optimal prodrugs for the carboplatin family, as the conjugates seem to be neutralized during the endocytic process and do not manage to effectively reach the nuclear DNA. In a more successful example, a platinum-(IV) complex *c*,*c*,*t*-[Pt(NH_3_)_2_Cl_2_(O_2_CCH_2_CH_2_CO_2_H)-(O_2_CCH_2_CH_2_CONH-PEG-FA)], containing a folate derivative (FA) at an axial position, was synthesized and attached to the surface of a single-walled carbon nanotube (SWNT-PL-PEG-NH_2_) (Figure 5A) and subsequently evaluated in vitro for its anti-tumor activity in FR-positive human choriocarcinoma (JAR) and human nasopharyngeal carcinoma (KB) cell lines [71]. As in the previous example, the Pt-(IV)-SWNT construct entered the cells through FR-mediated endocytosis [71]. Once inside the cell, the Pt-(IV) was reduced to Pt-(II), causing the release of cisplatin. Cisplatin subsequently accumulated in the nucleus, where it caused the formation of platinum-DNA adducts and triggered the intrinsic cell death pathways. Importantly, Dhar and co-workers demonstrated that the Pt-(IV)-SWNT construct was eight times more toxic compared to cisplatin in the FR-positive cancer cells [71]. In another study, a human epidermal growth factor receptor 2 (HER2) affibody-targeted Mal-PEG2000-DSPE micelle was mixed with cisplatin-loaded liposomes, composed of hydrogenated soy phosphatidylcholine/cholesterol/mPEG2000-DSPE, to generate HER2 affibody-targeted cisplatin liposomes [72]. It was seen that HER2^+^-SK-BR-3 cells, exposed to the generated cisplatin-loaded anti-HER2 affisome, exhibited higher platinum accumulation and displayed increased death at lower concentrations of the cisplatin-loaded anti-HER2 affisome compared to its liposome counterparts. Moreover, the cisplatin-loaded anti-HER2 affisome showed greater therapeutic efficiency compared to the non-targeted liposome in a HER2-positive mouse model. Even more promising was the observation that anti-HER2 affisome treatment also extended the overall survival of the mice [72].

Targeting the transferrin receptor underlines the therapeutic potential of specific ligand conjugations, as demonstrated with doxorubicin-loaded lipid-coated transferrin-conjugated nanoparticles that inhibit more efficient lung tumor growth compared to non-targeted nanoparticles [73] and with a dual-targeting transferrin conjugate that is able to penetrate the blood-brain barrier during glioma-targeting therapy [74].

### 4.5. Integrins

Integrins are dimeric cell adhesion receptors consisting of non-covalently associated α and β subunits. Their primary function is to mediate the attachment of cells to their extracellular matrix components or to other cells. Integrins play a crucial role in various biological processes including cell migration and proliferation [75,76]. It is well reported that the mesenchymal integrins αvβ3, αvβ6, and α5β1 are usually expressed at low or undetectable levels in normal adult cells but become highly upregulated in most cancer cells [75]. In adherent, cisplatin-resistant ELA cells and the non-adherent, cisplatin-sensitive EATC parental cell line, it has been demonstrated that the cisplatin-resistant cells had a significantly higher expression of integrin subunit α5, αV, and β1 compared to the cisplatin-sensitive cells [76]. Furthermore, it was found that cellular adhesion of ELA cells could be reduced by 40% in the presence of an Arg-Gly-Asp (RGD) peptide, which is commonly used as a competitive inhibitor of fibronectin binding [76]. More importantly, selective integrin β1 KD improved the cellular sensitivity of cisplatin-resistant ELA cells towards cisplatin, seen as an increased caspase activation, whereas it had no effect on cisplatin sensitivity in EATC cells [76]. This could indicate that the mesenchymal integrins αvβ3, αvβ6, and α5β1 might be decent targets for the selective recognition and delivery of Pt-based chemotherapy. In this context it is noted that Wang and colleagues designed RGD peptide conjugated αvβ3 integrin ligands conjugated with cyclic arginine-glycine-aspartic acid-tyrosine-lysine peptide (cRGDyk) and loaded them with cisplatin (RGD-CIS-liposome) or Pbs (RGD-PbS-liposome) (Figure 6A) [77]. Using mouse prostate (RM-1) cancer cells as an in vitro model system, they found that cellular liposome uptake was improved through cRGDyk and αvβ3 interaction and that RGD-CIS-liposomes showed higher cytotoxicity compared to CIS-liposomes (Figure 6B). The IC_50_ values for cisplatin, CIS-liposomes, and RGD-CIS liposomes were determined at 15, 10, and 2 µM, respectively, indicating that cisplatin-loaded RGD-CIS-liposomes enhance the therapeutic effect at low concentrations, presumably with fewer systemic side effects [77]. In vivo results revealed that treatment with RGD-CIS-liposomes inhibited osteoclastic and osteoblastic bone lesions, relieved pain, and delayed cell death [77]. Using CT scans and X-rays, Wang and co-workers also demonstrated that osteoclastic/osteoblastic lesions, seen in tumor-bearing hind limbs, were inhibited in mice treated with RGD-CIS-liposomes [77]. This was taken to indicate that RGD-conjugated liposomes might serve as an effective drug delivery system for targeted and synergistic therapy of bone metastases [77]. 

### 4.6. Passive Diffusion

Designing new platinum complexes equipped with physical and chemical properties that make them relay on passive diffusion for cellular delivery might be an alternative strategy to overcome platinum drug resistance caused by decreased intracellular accumulation. It is well known that passive diffusion mainly depends on the lipophilicity and overall charge of the compound. A well-described platinum compound, which mainly relies on passive diffusion, is the cisplatin analogue satraplatin (JM-216); *trans*,*cis*,*cis*-bis(acetato)amminecyclohexylamine dichloroplatinum(IV) [78]. Satraplatin was designed such that the stability and lipophilicity were appropriate for oral administration and gastrointestinal absorption and identified through preclinical studies as a possible candidate to overcome cisplatin resistance in osteosarcomas and ovarian cancer [78,79]. Once circulating in the bloodstream, satraplatin undergoes reduction to six distinct platinum-(II) species. Similar to cisplatin, satraplatin mainly mediates its effects through the formation of DNA cross-links, DNA breaks, and subsequent inhibition of DNA transcription [78]. In several preclinical studies, satraplatin displayed a better toxicity profile than cisplatin and has even showed promising activity in cisplatin-resistant cell lines [78]. The capability of satraplatin to overcome cisplatin resistance is thought to arise from its ability to introduce bulkier DNA lesions compared to cisplatin, which might be more difficult for the cells to remove and tolerate [79]. Satraplatin has gone through several clinical trials and shown promising clinical activities in patients with prostate, ovarian, cervical, and non-small lung cancer [78,79].

**Figure 7:**
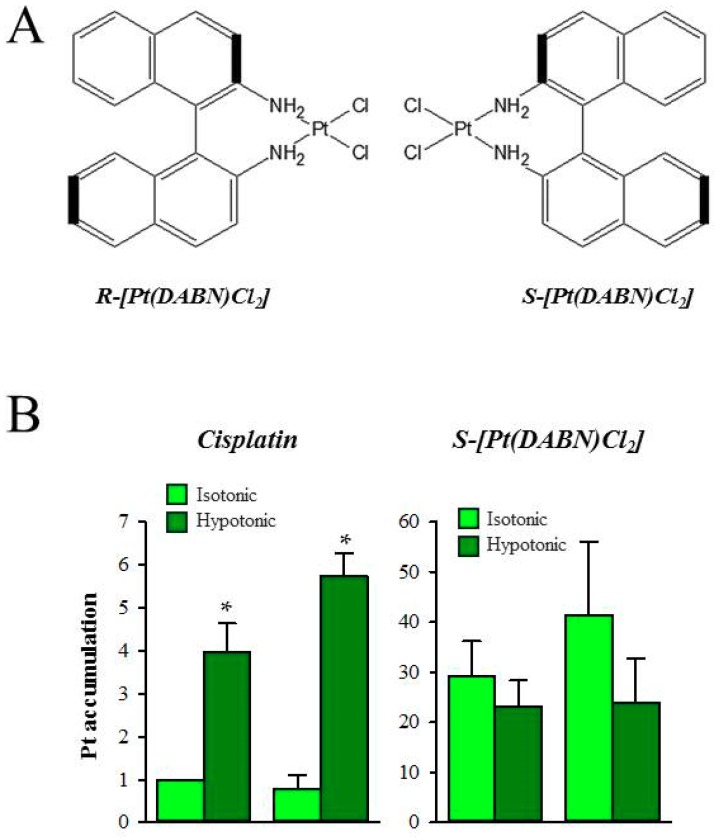
Cellular uptake of Pt enantiomeric complexes *S*-Pt(DABN)Cl_2_ and *R*-Pt(DABN)Cl_2_. (**A**) Cisplatin, *R*-DABN-Pt(II)Cl_2_, and *S*-DABN-Pt(II)Cl_2_; and (**B**) Effect of hypotonic exposure (4 h) on the cellular accumulation of 50 µM cisplatin or *S*-[Pt(DABN)Cl_2_] measured by the flameless AAS with deuterium compensation. * Indicates that uptake in hypotonic medium is significantly increased compared to uptake in isotonic medium. Figures adapted from [80].

The anti-cancer activity of two enantiomeric *R*- and *S*-1,1’-binaphthyl-2,2’ -diaminodichlorido-Pt(II) complexes (*S*- and *R*-[Pt(DABN)Cl_2_]) (Figure 7A) in cisplatin-sensitive (GUMBUS) and cisplatin-resistant (CDDPrGB) non-Hodgkin B-cell lymphoma cells have recently been evaluated [80]. *S*- and *R*-[Pt(DABN)Cl_2_] were investigated for the first time by Bombard et al., who demonstrated that the two enantiomers are able to overcome cisplatin resistance in human ovarian A2780 and colorectal HCT116 cell lines [81]. Furthermore, they demonstrate that the S-isomer seemed to be more potent than the *R*-isomer [81]. Using reverse-phase HPLC to evaluate the lipophilicity of the compounds, it was demonstrated that the two enantiomers were less hydrophilic compared to both cisplatin and oxaliplatin [82]. As the cellular platinum accumulation has been described to be exponentially related to the lipophilicity of the compounds, it was assumed that the two enantiomers have similar uptake, which is higher than that of both cisplatin and oxaliplatin [82]. Similar to findings by Bombard and colleagues [81], later work indicated that the CDDPrGB cells are not cross-resistant to neither *S*-[Pt(DABN)Cl_2_] nor *R*-[Pt(DABN)Cl_2_] [80]. Based on the IC_50_ values, *S*- and *R*-[Pt(DABN)Cl_2_] were determined to be more active compared to cisplatin in both GUMBUS and CDDPrGB cells [80]. As for the previous findings, the *S*-isomer seemed to be more active compared to the *R*-isomer, which may indicate that the chirality of the two compounds influences their biological activity. Investigating the time course for cellular accumulation, it was found that the cellular accumulation of the two enantiomers is rapid and reaches a plateau within two hours of exposure in both GUMBUS and CDDPrGB cells [80]. In contrast, the cellular accumulation of cisplatin is rather slow and does not reach equilibrium within an observed eight-hour period. The cellular accumulation of *S*- and *R*-[Pt(DABN)Cl_2_] turned out to be significantly larger compared to cisplatin but insensitive to hypoosmotic exposure, which is known to activate the volume-sensitive, LRRC8A/D-dependent VRACs and facilitate cisplatin accumulation (Figure 7B). The cellular accumulation of the two enantiomers may therefore involve a transport mechanism independent of the LRRC8A/D-dependent VRACs. Due to the high lipophilicity, it is assumed that the cellular accumulation of the two compounds may involve passive diffusion across the lipid bilayer. Finally, it turned out that the cellular action of the two enantiomers similar to cisplatin involves G0/G1 cell cycle arrest and apoptotic cell death, induced by activation of the extrinsic and intrinsic apoptotic pathways, i.e., activation of Noxa, caspase-9, p53, and Bid, as well as inactivation of PARP [80]. The clinical effectiveness of lipophilic drugs, unless they are caged, can be questioned as the benefit of increasing lipophilicity, which increases cellular uptake and cytotoxicity, might be outweighed by reduced penetration in tumors.

## 5. Concluding Remarks

The design of new Pt-based drug candidates is a challenging task. Several pharmacokinetic parameters have to be taken into consideration, e.g., renal clearance, solubility (hydrophilic vs. lipophilic), metabolic half-life, bioavailability, protein binding, and possible side effects. These are generally known as ADME-Tox parameters (absorption, distribution, metabolism, excretion, and toxicity). The next challenge is to develop solid pre-clinical model systems that mimic the tumor microenvironment to test the relevance of information obtained with 2D cultures of wild-type/drug-resistant cell lines.

Facing the unwanted development of drug resistance, new drugs exploiting various facilitating uptake mechanisms are being developed and evaluated for their putative anti-tumor activities. As resistance to cisplatin often involves reduced expression/activity of Cu-transporters and/or the volume sensitive osmolyte transporters (VRACs), the emphasis has been on new Pt-based, chemotherapeutic nanocarriers (liposomes, nanoparticles, nanotubes) conjugated with specific ligands (PEG, transferrin, folate) to specifically target tumor cells overexpressing the appropriate receptors. Unfortunately, the antitumor efficiency of nanocarriers relies on receptor-mediated endocytosis, intracellular lysosomal degradation, and release of the drug in an active form, which might limit their therapeutic use. The lipophilic Pt-based agents may be suitable for oral administration, as they are easily absorbed through the intestinal mucosa cells by passive diffusion. However, these compounds might also have a tendency to accumulate in adipose tissue, which limits their excretion. Water-soluble compounds, in contrast, may not be suitable for oral administration, as these compounds mostly rely on the presence of a drug transporter or channel in the plasma membrane of the intestinal mucosa cells. Moreover, the bioavailability of these compounds may be lower due to high renal clearance and detoxification.

Designing Pt-based conjugates or dual-drug co-delivery nanocarriers with a balanced ratio of drugs that are capable of inducing cell death through multiple intracellular mechanisms and at the same time selectively targeting rapidly dividing cancer cells (through specific biomarker recognition and targeting) may be a strategy to overcome the development of chemotherapeutic resistance and minimize systemic toxicity. However, some caution should be employed as the resistance mechanisms seen in experiments on resistant cell lines might have questionable relevance to the multifactorial resistance seen in solid tumors and the gain that EPR provides might be outweighed by the costs of lower tumor penetration by large entities.
